# Sign learning and its use in a co-enrollment kindergarten setting

**DOI:** 10.3389/fpsyg.2022.920497

**Published:** 2022-09-08

**Authors:** Madlen Goppelt-Kunkel, Anne Wienholz, Barbara Hänel-Faulhaber

**Affiliations:** Department of Special Education, Faculty of Education, Universität Hamburg, Hamburg, Germany

**Keywords:** sign language, language learning, co-enrollment, kindergarten, language development delay, deaf

## Abstract

Experimental studies report positive effects of signing for language acquisition and communication in children with and without language development delays. However, little data are available on natural kindergarten settings. Therefore, our study used questionnaire data to investigate the sign learning in hearing children (aged 3;7–5;9 years) with and without language development delays in an inclusive kindergarten group with a co-enrolled deaf child (aged 3;8  years) and a deaf signing educator. We observed that the hearing children in this co-enrollment group learned more signs than the hearing children from groups with only hearing educators who learned signs in a training program. Hearing children’s sign learning showed a tendency toward correlating positively with their level of spoken language development. However, the individual background for children with language development delays impacted this relationship. Additionally, we examined the modality use of all children in interactions with hearing and deaf educators and peers using questionnaire and video data. Despite acquiring signs, hearing children predominantly used spoken language with hearing educators and predominantly nonverbal communication strategies with the deaf educator and the deaf child. Children with language development delays used code-blending with hearing educators in a few cases. The deaf child used mainly sign language for interactions with the deaf educator and mainly nonverbal communication with hearing educators and peers. Overall, our results suggest that the presence of a deaf educator increases sign learning in hearing children. However, in interactions during free play, they barely used signs making it particularly challenging for the deaf child to participate. This reveals that, in addition to a deaf role model, more sign language competent peers and targeted approaches increasing the use of the visual modality are required.

## Introduction

Communication skills are key to participating in interactions (Guralnick et al., 1996, 2006; Odom et al., 2002). Interactions, in turn, are related to other areas of development, such as social–emotional skills, cognition and language (see DeLuzio and Girolametto, 2011). Deaf children and children with delayed spoken language abilities are at risk of being excluded from interactions (Odom et al., 2002; Preisler et al., 2002). For deaf children, communication in sign language offers the possibility to take part in verbal interactions (Marschark et al., 2006). Moreover, for hearing children with and without language development delays, studies reported that signs or gestures can increase communicative abilities (Bonvillian et al., 1981; DiCarlo et al., 2001). Consequently, the extent to which the visual modality is used by all group members in interactions is particularly crucial for the development of deaf children and children with spoken language development delays. Therefore, our study focuses not only on sign language learning but also on its use in a co-enrollment kindergarten setting with a deaf child and hearing children with and without language development delays.

### Sign learning in deaf and hearing preschool children

Deaf children of deaf parents acquire sign language from their parents as native languages and reach similar milestones at similar ages compared to hearing children acquiring spoken languages (Chen Pichler, 2012). This access to a perceptible language offers age-appropriate language, cognitive, and social–emotional development (Marschark et al., 2006). Besides studies reporting positive effects of signs or gestures on spoken language acquisition in children with various profiles, several studies investigated the extent to which signs are used to communicate by different target groups. These studies were based on the hypothesis that children could possibly bypass or compensate for certain abilities necessary for spoken language *via* the visuospatial modality (Bonvillian et al., 1981; Kay-Raining Bird et al., 2000). Already many decades ago, it was suggested that the motoric, visual, and kinesthetic skills in children with autism are more advanced compared to their auditory-verbal ones (O’Connor, 1971). Moreover, for children with Down syndrome, studies have shown that their visual memory is more developed compared to their auditory memory (Kay-Raining Bird and Chapman, 1994). In fact, studies found some children with autism to acquire signs faster than spoken words (for an overview, see Bonvillian et al., 1981; Nunes, 2008). Additionally, children with Down syndrome and typically developing children learned novel words better if the words were presented in parallel with signs than when words or signs were presented separately (Kay-Raining Bird et al., 2000). Producing intelligible spoken language often presents a motor challenge for children with Down syndrome while signing requires less precise fine motor skills (Kay-Raining Bird et al., 2000). Based on this observation, signing is offered to hearing babies and young children to provide another way of communication until fine motor skills are developed (Kay-Raining Bird et al., 2000). Therefore, signs are sometimes offered to children in kindergarten settings but only a few studies on sign learning in hearing children with and without disabilities are available.

A study by DiCarlo et al. (2001) assessed the spoken language development and sign learning in toddlers with and without disabilities (aged 1;3-3;0 years) of two inclusive classrooms in which signs had been implemented in hearing teacher-child interactions. They observed that some signs were learned while the development of spoken language was not inhibited. Wijkamp et al. (2010) investigated the sign learning of children with severe language development delays (aged 2;6–4;7 years) in a setting where Sign Supported Dutch was used by the hearing educational staff and therapists. They found some children to have learned single signs, especially when there was little auditory-vocal communication. A recent larger study by Schüler et al. (2021) investigated the sign learning of children with and without language development delays in inclusive kindergarten groups. The hearing educators in these groups participated in a training program familiarizing them with signs and their use. Six months after implementing the sign training sessions, Schüler et al. (2021) reported a significant increase in the children’s sign vocabulary. However, language role modeling impacted children’s sign acquisition significantly: in groups with hearing teachers using many signs, children’s vocabulary was significantly larger than in groups where signs were hardly implemented (Schüler et al., 2021). Moreover, sign learning correlated positively with spoken language development: children with better language skills learned significantly more signs than children with lower language skills.

### Sign use in deaf and hearing preschool children in interactions

Concerning interactions, there are studies from different kindergarten settings often focusing on different aspects such as educator–child or peer interactions, children’s language profiles, and language modality. Modality is the channel through which language is produced and received, i.e., spoken languages *via* the auditory-vocal modality and sign languages *via* the visual-gestural modality (Meier et al., 2009). In kindergarten settings, where only spoken language is used, it is particularly challenging to join interactions for children with language development delays or hearing loss (Guralnick et al., 1996; Odom et al., 2002; Preisler et al., 2002; DeLuzio and Girolametto, 2011). Therefore, some studies investigated the effect of sign use by hearing educators on children’s modality use in educator-child interactions. The hearing children with and without disabilities in DiCarlo et al.’s study showed increased communication in both speaking and signing in hearing educator-child interactions (DiCarlo et al., 2001). Wijkamp et al. (2010) observed an increase in the use of gestures or signs in educator-child interactions in some of the children with severe language development delays when they used less spoken language. The children’s individual background was driving this effect. Overall, the study observed a large increase in gestures but only a small increase in sign production within hearing educator-child interactions (Wijkamp et al., 2010). Preisler et al. (2002) investigated interactions between deaf children with cochlear implants and their educators in kindergarten settings for deaf and hard of hearing children (DHH) in which speech and signs were used. In these educator–child interactions, speech was used predominantly and often supported by signs. They reported that conveying linguistic content was more difficult when more speech without signs was used. But when the educators ensured eye contact with the children and clearly conveyed the context, the children understood simple instructions. In contrast, in deaf children with cochlear implants from a kindergarten setting for deaf children with educators using sign language, Preisler et al. (2002) observed extensive and abstract communication in educator–child interactions.

In terms of peer interactions, there are a few more studies available investigating communicative interactions between DHH children, children with language development delays and their typically developing hearing peers. Peer interactions are important for children to learn social skills and are critical contributors to social development (Corsaro, 1997, 2013; Antia et al., 2011). Paying attention to language development delays is particularly important as they were found to be a risk factor for being excluded from peer interactions in the auditory-vocal modality (Guralnick et al., 1996, 2006; Odom et al., 2002). In kindergarten groups with only deaf children where sign language was used, Preisler et al. (2002) reported that deaf children with cochlear implants used sign language at an age-appropriate level in peer interactions. In contrast, for DHH children in an auditory-vocal mainstream setting, DeLuzio and Girolametto (2011) observed consistent with previous studies (Arnold and Tremblay, 1979; Antia et al., 1993; Minnett et al., 1994; Spencer et al., 1994; Brown et al., 2000) that they received fewer interaction requests from hearing peers than their hearing peers and that DHH children’s initiation attempts were responded to less frequently by their peers than for hearing children. Overall, many studies report that not only entering in interactions, but also maintaining them is challenging for deaf children in auditory-vocal settings (for an overview see Xie et al., 2014). However, study results differ depending on factors such as the children’s and their interlocutor’s language ability and mode of communication, their familiarity, or age (see Antia et al., 2011). In preschools for DHH children where spoken language with supporting signs was used, Preisler et al. (2002) found children without sign language competencies to communicate mainly using pointing, gestures, or eye-contact. Similarly, Antia et al. (1993) describe that hearing and DHH children using oral or total communication interact mainly using gestures, exchanging objects, or playing games without verbal communication with each other. Furthermore, children with developmental language delays or hearing loss are reported to communicate more with educators and less with peers in comparison to the age-matched hearing children (Kniel and Kniel, 1984; Odom et al., 2002; Antia et al., 2011). Interacting primarily with educators is also reported by Preisler et al. (2002) for deaf children attending a mainstream setting with a sign language competent assistant. These deaf children enrolled in mainstream kindergarten groups primarily interacted with the sign language competent assistant. In peer interactions during play, these deaf children were observed to take over only non-communicative roles. However, this study examined a setting with hearing sign language competent assistants that were primarily translating between sign and spoken language, rather than deaf professionals communicating in sign language in all interactions. In addition, due to the study design, most of the data were collected during activities initiated by adults, such as telling stories, but not during free play.

### Modality use in bimodal-bilingual kindergarten settings

In bimodal-bilingual settings, all children are offered sign and spoken language and, thus, two modalities are available for hearing and DHH children to communicate. A study by Schulz (2016) focused exclusively on peer interactions in a bimodal-bilingual preschool group with hearing and DHH children. Although children were reported to use both modalities, most children could be assigned to a specific language group (Schulz, 2016): The hearing children communicated predominantly with other hearing children in spoken language, whereas the DHH children communicated predominantly with other DHH children in sign language. Preferring peers with the same hearing status for interaction is consistent with previous research (e.g., Arnold and Tremblay, 1979; Antia et al., 1993; Minnett et al., 1994; Spencer et al., 1994; for an overview see Antia et al., 2011). Ardito et al. (2008) similarly reported sign language use in deaf and hearing children in a bimodal-bilingual preschool group, where hearing educators used spoken language accompanied with signs and deaf educators used sign language. They suggested that the deaf and hearing children not only showed progress in their literacy development, but also developed better skills in both languages, signed and spoken. To the best of our knowledge, there is no study examining modality use in peer interactions in a bimodal-bilingual setting with both deaf and hearing educators, but with only one deaf child. The question arises how communication is shaped when only one deaf child is present in a bimodal-bilingual setting. Moreover, there is a lack of studies examining sign learning and its use in hearing children with and without language development delays when a deaf educator acts as a sign language role model. A co-enrollment setting of deaf and hearing children, especially including children with language development delays, offers a unique opportunity to investigate language and communication in all children. But to what extent do children learn and use signs in a setting with a deaf educator and a deaf child? How do factors such as a child’s level of spoken language development affect sign learning and its use in interactions during free play? Free play is an important setting for the participation in social peer interactions which are essential during child development (Corsaro, 1997). However, there is hardly any data available so far for learning settings of social and cultural processes (Corsaro, 2013) in a co-enrollment setting with a deaf child.

Therefore, our study used questionnaire and video data to examine the sign learning and use of children in an inclusive kindergarten group into which a deaf child was co-enrolled simultaneously with a deaf educator six months before data collection, so that a bimodal-bilingual setting was established. In this study, a bimodal-bilingual setting refers to an environment in which at least one deaf educator communicates with all children in sign language while at least one hearing educator communicates in spoken language, accompanied in part by signs. The children in our sample are supervised by one deaf educator and several hearing educators. Our data will be directly contrasted with data from inclusive kindergarten groups with hearing educators of Schüler et al. (2021). This comparison of sign learning in children with and without language development delays under different input conditions allows for a more extended assessment of role modeling as an influential factor on children’s sign learning. Following Schüler et al. (2021), we also investigated the correlation between hearing children’s spoken language abilities and their sign learning. In addition, the data are analyzed to determine the use of different modalities in interactions during free play in order to assess which participation opportunities such a co-enrollment setting offers to children with different language learning prerequisites.

## Materials and methods

### Participants

In total, 12 children (seven boys and five girls) from a co-enrollment kindergarten group participated in this study (for detailed participant background information, see Table 1 in the results section). Eleven children were hearing (age range = 3;7–5;9 years, *M* age = 4;5 years). Eight of them were monolingual native German speakers while three were bilingual with German as one of their languages. One child (age = 3;8 years) was a third generation deaf native signer acquiring German Sign Language (DGS) from birth from her deaf parents. All children included in the study had been attending the group for at least six months with the deaf child being co-enrolled in the group exactly six months ago. At that time, a deaf signing educator exposed to signs since the age of six years and using DGS as primary mode of communication since the age of 12 years, was employed in the group in parallel. Three hearing children received at least one kind of therapy like speech therapy, ergotherapy or physical therapy, but mostly in combination. Of these three children, one child was diagnosed with epilepsy affecting the speech center, one child had been described as having three detected genetic defects influencing cognitive and motoric development and one child had no diagnosed disability but showed a language delay in German and received ergotherapy. Additional six children of the same preschool group were excluded from the study due to enrollment of less than six months prior to data collection (*n* = 5) or with complex disabilities preventing the acquisition of sign or spoken language (*n* = 1). Participants’ legal guardians provided written informed consent prior to participation in the study.

**Table 1:** Demographic data, SBE-3-KT-score and sign score of the children sorted by hearing level, age of German acquisition and speech therapy.

Child no.	Gender	Age in months	Individual background	Therapy	Language	SBE-3-KT score (max. 172)	Sign score (max. 94)	Speech intelligibility in %	Speech comprehension in %
*Hearing children without speech therapy and German acquisition from birth*									
1	M	44	–	–	German, Polish	118	2	60	70
2	F	45	–	–	German	153	17	75	90
3	M	50	–	–	German	172	9	90	100
4	F	52	–	–	German	172	30	100	100
5	M	54	–	Ergotherapy	German, Arabic	39	3	30	30
6	M	55	–	–	German	171	10	100	100
7	M	57	–	–	German	172	7	100	90
8	F	64	–	–	German	172	31	100	100
*Hearing child without speech therapy and successive acquisition of German*									
9	M	53	–	–	Croatian, English, German (starting at 3 months)	124	35	75	80
*Hearing children with speech therapy*									
10	F	43	Epilepsy (caused by FCD or ganglioglioma), speech center affected	Speech therapy, ergotherapy	German	6	17	50	90
11	M	69	Three detected genetic defects	Speech therapy, ergotherapy, physical therapy	German	144	0	70	70
*Deaf child*									
12	F	44	Sensory-neural deafness	Speech therapy, ergotherapy	DGS, German	–	93	10	0

### Materials

In this study, we used the combination of questionnaire and video recordings to tackle our research question. The questionnaire was answered by both parents and educators and comprised four different sections with demographic information being provided at the beginning of the questionnaire prior to the sections (see Supplementary Material A for the educator questionnaire). The first section assessed vocabulary knowledge in sign and spoken language by presenting parents and educators with a list of 94 words and signs and ask them to mark each word and sign that the child is actively producing as a word and as a sign separately. This list contained 82 items extracted from the German language screening test SBE-3-KT (Suchodoletz et al., 2011) and an additional set of 12 signs and their corresponding translation equivalents from Schüler et al. (2021) often used in kindergarten communication settings and, thus, allowing for a direct comparison of both studies. The second section included 15 items from the grammar section of the SBE-3-KT test to measure children’s language developmental status more extensively. The next section collected information on the use of modalities in interactions with educators and peers overall to analyze children’s pragmatic-communicative skills. These questions were adapted from the Pragmatic Profile by Dohmen (2009) with sign and sign language as additional response options. Finally, the last section required the rating of each child’s speech intelligibility and comprehension on a scale of 0–100 in full numbers to better assess their abilities in spoken communication.

In addition, video data were collected during free play sessions in order to analyze language and modality use during interactions. A total of 13 cameras were installed in the rooms so that the children’s interactions could be recorded in all areas as far as possible. One hour of free play was filmed for each of the two survey days per child.

### Analysis

#### Questionnaire data

The spoken vocabulary and grammar part was evaluated following the given procedure of the SBE-3-KT (Suchodoletz et al., 2011). We evaluated only the questionnaires completed by the educators as these are considered more reliable than the parents’ questionnaires because the educators are more familiar with the signs presented in the questionnaire and their answers are more closely related to the kindergarten setting. For each child, a sign score and an SBE-3-KT score was calculated. For the sign score, each sign of the list presented in section one of the questionnaire marked as used by the child more than once was assigned one point leading to a maximum score of 94. In the evaluation of the spoken language part, a total of up to 172 points could be achieved.

First, we compared the sign score of the 11 hearing children from our co-enrollment group with the data of Schüler et al. (2021) using similar materials. They analyzed the sign learning of 289 children from inclusive kindergarten groups with only hearing educators who were trained in using signs six months prior to data collection. The children were divided into two groups based on implementation strength: One group consisted of 145 children (age range = 2;1–6;3 years, *M* age = 4;4 years), whose educators used signs frequently, i.e., high implementation strength. The other group comprised 144 children (age range = 1;7–6;6 years, *M* age = 4;4 years), whose hearing educators used signs rarely, i.e., low implementation strength (for a detailed description of the participants demographic data see Supplementary Material B). As the Shapiro–Wilk test revealed no normal distribution of the data (*W* = 0.76, *p* < 0.001), we used a nonparametric Kruskal–Wallis to test for the effect of group. A *post hoc* Wilcoxon rank-sum test with the Holm method for value of p adjustment (Holm, 1979) was applied to compare the sign score of our co-enrollment group to the two implementation groups of Schüler et al. (2021). In parallel to Schüler et al. (2021), we investigated the correlation between the sign score and the SBE-3-KT score. For this purpose, we calculated the non-parametric Kendall’s rank correlation *τ* for all 11 hearing children displayed in a scatterplot in the results section.

Regarding the pragmatic profile, we only evaluated the four questions that concern the children’s active use of a modality during interactions to get an impression of children’s modality use over time from the educator’s perspective. The first three questions relate to educator-child interactions and the fourth question relates to peer interactions (for details, see the Supplementary Material A).

#### Video data

The video data were coded with respect to language use and interactions motivated by Preisler et al. (2002) and Schulz (2016) (see Supplementary Material C for further information on the coding scheme). Of all video data, 30 min of each child were coded from two survey days resulting in a total of 60 min of coded free play per child. All children were present on the same two survey days, except for children 9, 10, and 11. As child 10 and 11 were not present on one of these survey days, videos from another day were coded for these children. Child 9 was not present on this other day; thus, the child was excluded from the video analysis.

For each interaction, children’s interaction partners were determined, and modalities used by the children and their interaction partners were coded including spoken language, sign language, code-blending, code-switching, and nonverbal communication strategies such as pointing, nodding, head shaking, laughing, giving or taking objects. Nonverbal communication was only selected when no lexicalized words or signs occurred within the coded interaction and, thus, none of the other categories applied. Code-blending was selected when spoken words and signs were produced simultaneously, even if this happened only once within an interaction. In contrast, code-switching was assigned if a child switched from spoken language to sign language or vice versa. The videos were coded by two student assistants who are hearing advanced signers and had previously been trained in a similar coding scheme during a previous project (Goppelt-Kunkel et al., 2021). To determine consistency during coding, a reliability analysis was performed on 20% of the data from five randomly selected children, including the deaf child, using Cohen’s Kappa (Cohen, 1960). Coders show substantial agreement for coding used modalities (*κ* = 0.74, 95% CI [0.55, 0.94]).

## Results

### Sign score and SBE-3-KT score

Table 1 below presents an overview of the sign score and the SBE-3-KT score as well as additional demographic information for each child in the co-enrollment group.

**Figure 1:**
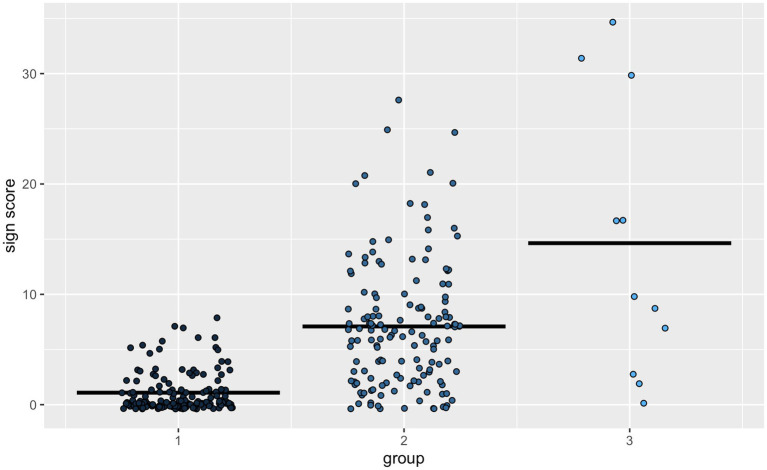
Sign score for hearing children by groups with (1) the inclusive kindergarten groups with low implementation of signs from Schüler et al. (2021), (2) the inclusive kindergarten groups with high implementation of signs from Schüler et al. (2021) and (3) the co-enrollment group with a deaf child and a deaf educator.

First, we assessed sign learning in the hearing children across all groups, i.e., in the co-enrollment group and the two implementation groups from Schüler et al. (2021) showing a main effect for group [*χ*^2^(2) = 130.51, *p* < 0.001, *η*^2^ = 0.43]. Follow-up tests comparing the low implementation group, i.e., group 1 (sign score *M* = 1.09), and the high implementation group, i.e., group 2 (sign score *M* = 7.09), each with our co-enrollment group, i.e., group 3 (sign score *M* = 14.64), revealed significant differences. The average sign score of the hearing children in the co-enrollment group was significantly higher than in implementation group 1 (*p* < 0.001, *d* = 0.39) and implementation group 2 (*p* = 0.05, *d* = 0.16) from Schüler et al. (2021) as shown in Figure 1.

**Figure 2:**
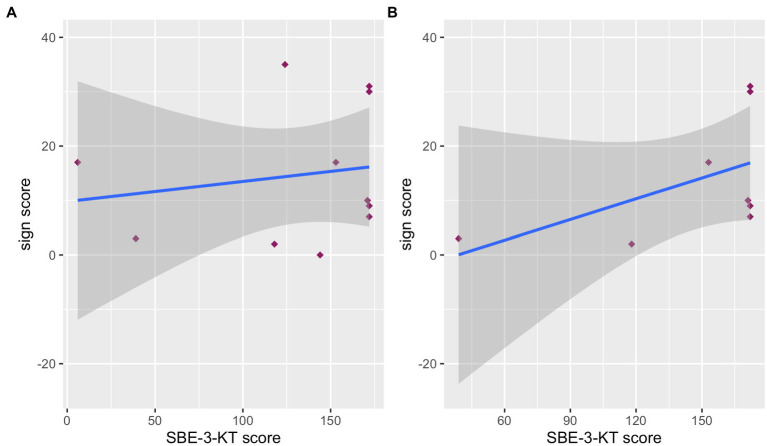
Correlation between sign score and SBE-3-KT-score for the co-enrollment group for (A) all hearing children and (B) only hearing children acquiring German from birth and without speech therapy.

Next, we analyzed the relationship between the sign score and the level of language development in spoken language in the hearing children of our co-enrollment group. Children’s sign score and SBE-3-KT score correlated weakly for the hearing children (*r_τ_* = 0.117, *p* = 0.630) but did not reach statistical significance. The data visualization in Figure 2A suggested that the two children undergoing speech therapy and the one child that did not acquire German from birth showed diverging patterns in the relationship between sign score and language development level. Therefore, we excluded these three children and repeated the correlation analysis (Figure 2B). When only including children, who learned German from birth and who did not show a language development delay (n = 8), the correlational coefficient increased to medium (*r_τ_* = 0.403, *p* = 0.184) but still the correlation did not reach significance even if a tendency toward significance can be observed. The lack of significance might be due to the very small sample size of our group and, thus, with a larger more homogenous sample significance might be reached. Nevertheless, the observed direction of the correlation in our data is consistent with Schüler et al. (2021) showing higher sign learning for children with advanced spoken language skills. Thus, irrespective of the tested group, a higher SBE-3-KT score seems to condition a higher sign score.

### Pragmatic profile

In educator-child interactions, hearing children without speech therapy and acquiring German from birth were all reported to use spoken language in complete sentences with some exceptions of single words, two-word sentences, or other modalities (for a summary presentation of the data, see Table 2). Child 4 additionally used sign language in one of the three queried situations. Child 5 did not use full sentences, but single words, two-word combinations or nonverbal communication strategies. Child 7 used, depending on the situation, complete sentences, single words or two-word combinations, signs, or used no language, but reacted emotionally with crying or anger. The two hearing children undergoing speech therapy used diverging modalities for communication: Child 10 used nonverbal communication strategies, reacted with emotions, used single signs, or single words depending on the communication context. Child 11 used single words or pointed to a desired object. The deaf child used sign language.

**Table 2:** Active use of modalities and communication strategies for each child in interactions with educators as indicated in the respective section of the educator questionnaire.^+^

Child	Spoken language	Sign language	Spoken language and sign language	Nonverbal communication (e.g., mimic or gestures)	Emotional/passive reaction (e.g., crying)
Child 1	+	−	−	+	−
Child 2	+	−	−	−	−
Child 3	+	−	−	−	−
Child 4	+	+	−	+	−
Child 5	+	−	−	+	−
Child 6	+	−	−	+	−
Child 7	+	+	−	+	+
Child 8	+	−	−	+	−
Child 9	+	−	−	+	−
Child 10	+	+	−	+	+
Child 11	+	−	−	+	−
Child 12	−	+	−	−	−

A “^+^” indicates that the modality was selected for the child for at least one question.

For participating in peer interactions, hearing children without speech therapy and acquiring German from birth were all reported to use spoken language (for a summary presentation of the data, see Table 3). Additionally, child 8 used a combination of words and signs and child 4 played next to other children without communicating with them. Child 9 that did not learn German from birth also communicated in spoken language in peer interactions. Child 10 who was undergoing speech therapy due to epilepsy used a combination of words and signs for peer interactions or played parallel to other children without communicating with them. Child 11 who was undergoing speech therapy due to genetic defects used spoken language for peer interactions or played by himself, thus without communicating with other children. The deaf child was reported to use indistinct spoken language toward peers for playing and to need help by educators to join peer interactions.

**Table 3:** Active use of modalities and communication strategies for each child in peer interactions.^+^

Child	Spoken language	Sign language	Spoken and sign language	Playing alone	Playing alongside the other children	Watching the other children	Need of adult guidance
Child 1	+	−	−	−	−	−	−
Child 2	+	−	−	−	−	−	−
Child 3	+	−	−	−	−	−	−
Child 4	+	−	−	−	+	−	−
Child 5	+	−	−	−	−	−	−
Child 6	+	−	−	−	−	−	−
Child 7	+	−	−	−	−	−	−
Child 8	+	−	+	−	−	−	−
Child 9	+	−	−	−	−	−	−
Child 10	−	−	+	−	+	−	−
Child 11	+	−	−	+	−	−	−
Child 12	+	−	−	−	−	−	+

A “^+^” indicates that the educators observed the respective modality.

### Video data

Overall, 1,254 interactions of all 11 children were coded with 193 educator-child interactions (15.4%) and 1,061 peer interactions (84.6%). Table 4 below provides an overview of all interactions separated by used modality and interaction partner.

**Table 4:** Occurrences of language modalities as percentages with absolute numbers in parentheses used by the hearing children and the deaf child when interacting with hearing educators, the deaf educator, hearing peers or the deaf peer in the video data.

Interactions	Spoken language	Nonverbal communication	Code-blending	Code-switching	Sign language	Total
*Hearing children*	*93.2% (1169)*
Hearing educator	54.3% (63)	42.2% (49)	3.4% (4)	0% (0)	0% (0)	9.9% (116)
Deaf educator	23.1% (3)	76.9% (10)	0% (0)	0% (0)	0% (0)	1.1% (13)
Hearing peer	72.4% (742)	27.4% (281)	0.2% (2)	0% (0)	0% (0)	87.7% (1025)
Deaf peer	13.3% (2)	80.0% (12)	0% (0)	0% (0)	6.7% (1)	1.3% (15)
*Deaf child*	*6.8%(85)*
Hearing educator	4.3% (1)	43.5% (10)	8.7% (2)	8.7% (2)	34.8% (8)	27.1% (23)
Deaf educator	0% (0)	36.6% (15)	0% (0)	0% (0)	63.4% (26)	48.2% (41)
Hearing peer	14.3% (3)	66.7% (14)	0% (0)	0% (0)	19.0% (4)	24.7% (21)

Hearing children were involved in 1,169 interactions and mostly interacted with their peers and less with the educators except for child 10, who was undergoing speech therapy. In interactions with hearing educators, the hearing children primarily used spoken language followed by nonverbal communication strategies and rarely code-blending. However, the two hearing children undergoing speech therapy, children 10 and 11, mainly used nonverbal communication strategies with their educators followed by spoken language. The four code-blending interactions were all observed in children with delayed spoken language development in German toward hearing educators, two in child 10, one in child 11 and one in child 5. When communicating with the deaf educator, the hearing children relied on nonverbal communication strategies but less on spoken language. In peer interactions, hearing children used predominantly spoken language followed by nonverbal communication strategies. In contrast, the children undergoing speech therapy mainly used nonverbal communication strategies for peer interactions but also spoken language. Code-blending and sign language were only applied in a few cases by hearing children while code-switching was not observed. The two peer-interactions with code-blending were observed in two hearing children without disabilities, child 7 and 8, both with child 4 without disabilities. The only observed interaction in which sign language was used by a hearing child was detected in child 5 when communicating with the deaf child.

The deaf child participated in 85 interactions predominantly with the educators and comparatively less with her peers. Communicating with educators, the deaf child predominantly interacted with the deaf educator mostly using sign language and, in fewer cases, nonverbal communication strategies. In contrast, interactions with the hearing educators were much less and the pattern of used modalities was reversed with additional observed modalities. The deaf child primarily used nonverbal communication strategies and sign language whereas spoken language, code-blending, and code-switching were only used in a few interactions. In interactions with her hearing peers, the deaf child applied mostly nonverbal communication strategies but also used sign language and spoken language.

## Discussion

In our study, we investigated sign learning in hearing children and language modality use of hearing children and a deaf child in a co-enrollment kindergarten setting. The deaf child was co-enrolled six months before data collection in parallel with a deaf educator. We observed that hearing children in the co-enrollment setting had learned significantly more signs than children from inclusive day care centers whose hearing educators had learned signs in a training program (Schüler et al., 2021). Children with more advanced spoken language skills demonstrate a tendency to higher sign scores except for children with certain individual backgrounds or later acquisition of German, although this observation did not reach significance possibly due to the small sample size. In interactions during free play, however, hearing children used predominantly spoken language. The deaf child used predominantly sign language with the deaf educator and predominantly nonverbal communication strategies with hearing educators and her peers. Code-blending was observed only occasionally, mostly by children with language development delays when communicating with hearing educators. Code-switching, on the other hand, was observed sporadically only in the deaf child when communicating with hearing educators.

### Sign learning

The analysis of sign learning revealed that the hearing children in our co-enrollment group with a deaf educator learned signs. Compared with data from children of inclusive day care groups with sign-trained hearing educators (Schüler et al., 2021) children in our co-enrollment group showed a significantly higher sign score. This difference seems not to arise due to different language development levels of the children, since the children from Schüler et al.’s groups with low sign scores showed the highest spoken language skills whereas the hearing children from the co-enrollment group showed the lowest spoken language skills. The major difference between our co-enrollment group and Schüler et al.’s groups is the presence of a deaf educator. The deaf educator and the deaf child are communicating predominantly using sign language, and, therefore, the co-enrollment group is exposed to the signed modality more extensively presumably leading to increased sign learning. This suggests that hearing children might learn more signs due to deaf role models. However, we cannot exclude previous occasional sign contact since the deaf child attended another group of this kindergarten before enrollment in the observed group and another deaf child in a different group attended the kindergarten two years ago. But, comparing the sign score of the co-enrollment group with the sign score of the children in the groups of hearing sign trained educators after 18 months of exposure in the data of Schüler and Hänel-Faulhaber (n.d.) suggests that even with prolonged contact with signs by hearing educators children seem to learn less signs as when a deaf role model is present.

Furthermore, we observed that hearing children’s sign learning showed a tendency to correlate positively with their spoken language abilities. However, this relation does not become significant in our data. Nevertheless, applying the analysis to a more homogeneous group by excluding children with language development delays and onset of German acquisition later than birth, the relation increases. The general tendency of this relation is in line with Schüler et al. (2021) who show that children with more advanced spoken language skills learned more signs than children with less advanced spoken language skills. The lack of significance might be due to the small sample size or the used test to assess the spoken language abilities as all hearing children are almost at ceiling. The SBE-3-KT was selected to compare our data with the data of Schüler et al. (2021), but for a more accurate calculation of the correlation between sign learning and spoken language abilities tests normed for the investigated age group are required. Nevertheless, this correlation is also reported for unimodal bilingual preschool settings and explained by increasing abilities to process language (*cf.*, Wode, 2009). However, in our small and heterogenous sample, we observed children with different patterns: The child with epilepsy, whose speech center was affected, had learned more signs than all other hearing children on average despite the lowest score in the spoken language test. This suggests that the child was able to use unaffected brain areas for language *via* the visual modality. This is supported by similar observations in children with Down syndrome (Kay-Raining Bird and Chapman, 1994; Kay-Raining Bird et al., 2000) and with autism spectrum disorders (O’Connor, 1971). Thus, a visual language provided the opportunity for more and improved communication with implications for other areas of development such as social–emotional and cognitive skills. However, the other child undergoing speech therapy did learn no sign from the tested list. This reveals that sign learning may depend on the individual background of a child with language development delay. The one child that did not learn German from birth and showed no age-appropriate language skills in German, had learned most signs of the tested list. Therefore, we assume that this child also benefited in a special way from the visual modality. It might be the case that the visual modality offered the opportunity to see aspects of the referents represented iconically (Lüke and Ritterfeld, 2014; Vogt and Kauschke, 2017). However, the other bilingual children with delayed language development in German learned fewer signs than all other hearing children in the co-enrollment group. This suggests that only some bilingual children show a preference for learning signs which might be driven by the age of acquisition of the national language as found by Schüler and Hänel-Faulhaber (n.d.). However, the child with successive acquisition of German of our co-enrollment group started to acquire German at the young age of three months, thus, being exposed to three languages or other factors might have been more crucial for his high sign score. Overall, our co-enrollment group’s heterogeneity provided the opportunity to observe different patterns of sign learning and modality use, but our findings need to be investigated in more depth in future studies with a larger and more controlled group of children.

### Modality use in interactions

Despite acquiring signs, hearing children in our study rarely used the visual modality in interactions and mostly interacted in spoken language instead. This finding is consistent with research from inclusive kindergarten groups whose educators were trained to use signs in interactions with children (Goppelt-Kunkel et al., 2021). Similar findings were reported from monomodal-bilingual kindergarten settings showing that children mostly communicate in the national language unless there is a need to use the second language offered (*cf.*, Wode, 2009).

In interactions with educators, two hearing children without disabilities sometimes used signs according to the questionnaire data, but in the video data sign use toward educators by hearing children was observed only for hearing children with language development delays: They sporadically used code blending in interactions with a hearing educator. Furthermore, in interactions with hearing educators, hearing children predominantly used spoken language. This finding is not surprising, as spoken language is the national language and the main mode of communication for both. In contrast, when communicating with the deaf educator, hearing children were never observed to use sign language or signs but mainly used nonverbal communication strategies and sometimes spoken language. This observation might be surprising because children at this age are expected to be aware of which language their interaction partners use (Petitto et al., 2001), however, this was observed in children growing up bimodal-bilingually from birth. In contrast, factors like language dominance and sociolinguistic context induce the use of the national language for active communication in bilingual children irrespective of their interlocutors´ language (Paradis and Nicoladis, 2007; see Müller et al., 2011 for an overview). This is reported for children acquiring a second language in a monomodal bilingual kindergarten setting as well (Wode, 2009). Our findings are in line with these observations. Additionally, it might be the case that the hearing children just do not have sufficient sign language skills yet to use signs in interactions.

In peer interactions, signs were barely used by hearing children as well: According to the questionnaire data, only two hearing children used a combination of words and signs, the child with language development delay due to epilepsy and one child without disabilities and growing up monolingually. This reveals that the child with epilepsy could communicate better with signs in some situations and, thus, could participate more easily in interactions. Therefore, this child benefited from the signs introduced in the group as indicated by richer sign than spoken vocabulary and the use of the visual modality for communication with both, educators and children. Using signs may have enabled that child to compensate for spoken language skills that were more challenging to acquire because of her individual background. Thus, a visual language may have been another way to participate in interactions. This assumption is additionally supported by the data from other children with language development delays who sporadically used mixed modalities in communication with hearing educators of our co-enrollment group and is consistent with Goppelt-Kunkel et al. (2021) as well. However, in peer interactions during the analyzed free play, the children undergoing speech therapy did not use signs at all but mainly nonverbal communication strategies and, rarely, spoken language. Perhaps, despite their limited spoken language skills, these children tried to communicate with the other hearing children in the language most used by them, the national language, consistent with observations in bilingual kindergarten settings (Wode, 2009). Additionally, please keep in mind that the child with epilepsy was present on only one of the two survey days when the deaf child was present, so that only half of the video data did allow for common interactions with the deaf child mainly interacting in sign language. The hearing child who was reported to use signs in peer interactions might use signs depending on its interaction partners. However, this is not observed in the video data. The only child that used sign language in a peer interaction with the deaf child in the video data was a different child receiving ergotherapy, growing up bilingually and showing a language development delay. This rare use of the visual–spatial language modality by hearing children makes almost all peer interactions between hearing children linguistically inaccessible to the deaf child.

The deaf child predominantly interacted with educators, especially with the deaf educator, consistent with the observations in Preisler et al. (2002) reporting this for mainstream settings with sign language competent assistants. For interactions with the deaf educator, the child predominantly used sign language, whereas with hearing educators, she predominantly used nonverbal communication strategies and somewhat less sign language. This suggests that the deaf child shows sensitivity to the educator’s language and, therefore, adapts to the educator’s language skills as described in Petitto et al. (2001). In peer interactions, according to the video data, the deaf child used mainly nonverbal communication strategies, occasionally sign language, and somewhat less frequently spoken language. Again, the deaf child presumably assesses the hearing peer’s language skills and chooses the most successful way to participate in interactions. With predominantly nonverbal communication, the peer communication behavior of the deaf child in the co-enrollment group less resembles bimodal-bilingual settings with DHH peers communicating in sign language but is rather comparable with observations from settings with speech accompanying signs or mainstream settings with hearing sign language assistants (Preisler et al., 2002; Antia et al., 2011; Schulz, 2016). But these settings lack age-appropriate language peers for deaf children to interact with. Thus, in addition to sign language input provided by educators, the presence of other DHH children seems to be crucial for the use of sign language in peer interactions with age-appropriate language (Spencer et al., 1994; Preisler et al., 2002; Ardito et al., 2008). Furthermore, the educators reported in the questionnaire that the deaf child needed assistance of adults to get involved in playing with other children and communicated in indistinct spoken language with them. The need for adult assistance to participate in peer interactions is consistently reported for deaf children in mainstream settings with sign language competent assistants (Preisler et al., 2002). So, six months after enrollment, the deaf child did not have equal chances to participate in interactions during free play as her hearing peers almost exclusively communicated in the auditory-vocal modality. Therefore, joining ongoing interactions seemed to be particularly challenging for the deaf child, even though it might be the case that some children already had prior knowledge of signing. As an additional factor, we need to consider that the deaf child was one of the youngest children in the group and peer-interaction is known to increase with age (Rubin et al., 1998). It must also be kept in mind that for DHH children in particular, time and familiarity with their peers seem to be important factors that might improve interactions (Lederberg et al., 1986; Kurkjian and Evans, 1988; Rodríguez and Lana, 1996). Nonetheless, more sign language competent peers are needed, as it is known from previous research that this is an important prerequisite for age-appropriate communication between peers (Preisler et al., 2002) also in a bimodal-bilingual setting (Ardito et al., 2008). Furthermore, language planning and modality planning, i.e., designated rooms or times in which communication is required exclusively in the visual modality, could increase the sign use of hearing children. On the one hand, this concept requires focused attention of the hearing children on the visual modality and, on the other hand, might lead to increased sign language skills in these children for peer interactions.

### Limitations

The heterogenous group in our study provided a unique opportunity to examine the sign learning and use of deaf and hearing children with and without disabilities in a bimodal-bilingual kindergarten setting with a deaf educator and hearing educators. But investigating this small co-enrollment sample also led to some limitations: The small sample size limited the statistical power of the comparison of the sign scores of the hearing children of our co-enrollment group with the sign scores of groups with only hearing educators from Schüler et al. (2021) as well as of the reported correlation exploring the relation between sign score and spoken language skills. Future studies should include a more homogenous group and an increased sample size.

Furthermore, the test used to assess the spoken language skills might not reflect individual differences in our data sufficiently. The SBE-3-KT was used to allow for a direct comparison with the data from Schüler et al. (2021), however, the test is normed for children from 32 to 40 months. Therefore, future studies with more age-appropriate spoken language assessments are needed in order to more accurately capture the relation between sign learning and spoken language abilities in bimodal-bilingual kindergarten settings.

Another restricting factor could be previous sign knowledge of the hearing children. It cannot be ruled out that some children had contact with signs prior to the hiring of the deaf educator since the deaf child attended another group within the same kindergarten before enrollment in the observed group. Moreover, another deaf child attended the kindergarten two years ago, but in a different group. However, data from Schüler and Hänel-Faulhaber (n.d.) suggest that time of sign exposure might have less impact on sign learning than sign language role modeling. They measured the sign learning of children with and without disabilities 18 months after their hearing educators were exposed to signs and observed a lower sign score than in our co-enrollment group indicating that longer exposure to signs does not lead to such effective sign learning as observed when sign language input is provided by a deaf educator.

Finally, it should be noted that the deaf child is one of the youngest children in the group and peer interaction is known to increase with age as mentioned above. To some extent, the lower number of interactions of the deaf child with other children could also be influenced by this fact. In addition, the deaf child attended the group for a shorter period of time than almost all other children studied. As outlined above, time and familiarity with peers are relevant for interactions of deaf children in particular, therefore, these factors might also have had an influence on the observed peer interactions.

## Conclusion

Overall, we observed that hearing children learned signs, but they barely used these for interactions, not even with deaf interlocutors. This suggests that more sign language input as well as language planning encouraging these children to use sign language are needed. Improving normally developing children’s sign language use in interactions is additionally important to increase opportunities for children who use sign language or signs for communication to participate in interactions: In our study, hearing children with language development delays used signs in restricted contexts. In particular for the deaf child, the fact that six months after co-enrollment hardly any signs were used in interactions during free play, especially between peers, limited the possibilities to participate in interactions. In addition to targeted approaches that strengthen the use of the visual modality, the presence of more deaf peers (Spencer et al., 1994; Ardito et al., 2008), and more deaf educators is required. Other factors should be kept in mind such as time to increase both familiarity between children and children’s sign language skills.

## Data availability statement

The original contributions presented in the study are included in the article/Supplementary material, further inquiries can be directed to the corresponding author.

## Ethics statement

Ethical review and approval was not required for the study on human participants in accordance with the local legislation and institutional requirements. Written informed consent to participate in this study was provided by the participants' legal guardian/next of kin.

## Author contributions

MG-K and BH-F contributed to the conceptualization and design of the study. MG-K conducted the investigation and wrote the original draft. MG-K and AW performed the formal analysis. MG-K, AW, and BH-F were involved in writing, reviewing, and editing of the manuscript. BH-F supervised and provided funding for the investigation. All authors contributed to the article and approved the submitted version.

## Funding

This study was funded by the German Federal Ministry of Education and Research (BMBF, FKZ: 01NV1706), awarded to BH-F and the Faculty of Education, Universität Hamburg, awarded to MG-K.

## Conflict of interest

The authors declare that the research was conducted in the absence of any commercial or financial relationships that could be construed as a potential conflict of interest.

## Publisher’s note

All claims expressed in this article are solely those of the authors and do not necessarily represent those of their affiliated organizations, or those of the publisher, the editors and the reviewers. Any product that may be evaluated in this article, or claim that may be made by its manufacturer, is not guaranteed or endorsed by the publisher.
