# Supplementary material for: Sign learning and its use in a co-enrollment kindergarten setting
Source: Front Psychol. 2022 Sep 8;13:920497. doi: 10.3389/fpsyg.2022.920497 (PMC9493243; doi:10.3389/fpsyg.2022.920497)
Supplement: Supplementary file 1 [file Data_Sheet_1.pdf]

SUPPLEMENTAL MATERIAL

Supplemental Material A: A translation of the educator questionnaire is provided here. The questionnaire contains questions about demographic information about the respective child and four sections assessing vocabulary (Part 1), grammar (Part 2), communicative skills, i.e., pragmatic profile (Part 3) and speech intelligibility and comprehension (Part 4).

Questionnaire on child language development

(for educational professionals)

General information

Child's name: ____

Gender: ☐ girl ☐ boy

Date of birth: ___ /___ /______

Today's date: ___ /___ /______

Name of kindergarten:

Name of kindergarten group:

Does the child receive any special educational support/therapy? ☐ yes ☐ no

If YES, please provide details in keywords:

Has the child been diagnosed with permanent hearing loss? ☐ yes ☐ no

If YES, please provide details below:

	right ear:	*left ear*:
Degree of hearing loss in dB		
Supply with hearing aid (HA) or cochlear implant (CI)	HA ☐ CI ☐ since______	HA ☐ CI ☐ since______

Additional information:

Does the child have physical disabilities, chronic diseases or other permanent impairments? ☐ yes ☐ no

If YES, please provide details in keywords:

Does the child grow up multilingual? ☐ yes ☐ no

If YES, please indicate all languages here:

If YES, since when has the child been learning German? *(Example: since 05/2014)*:

If the child grows up multilingual, please note that the information in the following questionnaire only refers to what the child says in **German.** In addition, **signs of German Sign Language** are recorded.

Important! The assessment should be carried out by a person who has known the child **very well for at least 3 months**. The first and second assessment should be done by the **same person**.

Part 1 - Vocabulary

Subsequently, you will find a list of words that young children typically have in their vocabulary.

There are a lot of words listed here as this is a collection of words used by different children and also by children who may be a bit older. So don't worry if the child only speaks a few of these words at this point. Go through the list and tick the words that the child **speaks and/or signs**.
However, only words/signs should be ticked that the child has used more than once and not those that it only repeats or understands. Please also tick words that the child pronounces slightly differently (e.g., "taufen" (German word for "baptize") instead of "kaufen" (German word for "buy") or "daußen" (German word for "outside" but with omitted "r") instead of "draußen" (German word for "outside"). If the child uses something similar (e.g., "cup" for "glass"), please write that next to the respective entry.

If the child uses both the word and the sign, please tick both.

	speaks	signs		speaks	signs		speaks	signs		speaks	signs
birthday	☐	☐	work	☐	☐	small	☐	☐	to run	☐	☐
girl	☐	☐	to water	☐	☐	to cook	☐	☐	with	☐	☐
to need	☐	☐	to say	☐	☐	outdoors	☐	☐	glass	☐	☐
head	☐	☐	tired	☐	☐	sand	☐	☐	bucket	☐	☐
to eat	☐	☐	ready	☐	☐	later	☐	☐	to like	☐	☐
throat	☐	☐	to laugh	☐	☐	music	☐	☐	clean	☐	☐
towel	☐	☐	to find	☐	☐	lamp	☐	☐	have to	☐	☐
spicy	☐	☐	finger	☐	☐	today	☐	☐	quiet	☐	☐
give a gift	☐	☐	wet	☐	☐	meat	☐	☐	to listen	☐	☐
light	☐	☐	new	☐	☐	umbrella	☐	☐	to fly	☐	☐
to clean	☐	☐	I	☐	☐	more	☐	☐	to play	☐	☐
to taste	☐	☐	nice	☐	☐	ear	☐	☐	now	☐	☐
breakfast	☐	☐	to buy	☐	☐	to lie (down)	☐	☐	paper	☐	☐
dirty	☐	☐	sun	☐	☐	carpet	☐	☐	warm	☐	☐
to cut	☐	☐	to live	☐	☐	fast	☐	☐	to jump	☐	☐
animal	☐	☐	to wait	☐	☐	cloud	☐	☐	to stand	☐	☐
chocolate	☐	☐	table	☐	☐	to wash	☐	☐	tooth	☐	☐
to tidy up	☐	☐	once again	☐	☐	to want	☐	☐	alone	☐	☐
cabinet	☐	☐	stone	☐	☐	tomato	☐	☐	water	☐	☐
toe	☐	☐	shoe	☐	☐	pen	☐	☐	stairs	☐	☐
away	☐	☐	room	☐	☐	heavy	☐	☐	street	☐	☐
full	☐	☐	soft	☐	☐	tongue	☐	☐	to search	☐	☐
to swim	☐	☐	to read aloud	☐	☐	meadow	☐	☐	to see	☐	☐
together	☐	☐	soup	☐	☐						

Part 2 - Grammar

1. Does the child already use word combinations/sentences of two or more words, ☐ yes ☐ no

such as "mommy book", "playing baby" or "Is that going in there?"?

If you ticked YES, please answer all subsequent questions. If you ticked NO, please continue to 'Part 3 – communication'.

In the following, always tick the option that **most closely** corresponds to what the child would say.

2.	☐ There cat.<br>☐ There's a cat.	3.	☐ Mommy shopping.<br>☐ Mommy is shopping.
4.	☐ This mine!<br>☐ This is mine!	5.	☐ Mommy cook.<br>☐ Mommy is cooking.
6.	☐ many car<br>☐ many cars	7.	☐ many flower<br>☐ many flowers

8th. Does the child use the sentence connector **and** ? (e.g., "I'll get the book **and** then you read it.") ☐ yes ☐ no

9. Does the child use the words my (grammatical gender male)/my (grammatical gender female) correctly? (e.g., **my** room, **my** doll, **my** toys) ☐ yes ☐ no

10 If the child doesn't want an apple, they're more likely to say:
☐ "Don't eat apple!"
☐ "I don't want to eat an apple!"

Does the child use the question word:

11.	**How?** – e.g., "How does game work?"	☐ yes	☐ no
12.	**What?** – e.g., "What have you got there?"	☐ yes	☐ no
13.	**Where?** - e.g., "Where's my ball?"	☐ yes	☐ no
14	**To where?** – e.g., "Where is Dad going?"	☐ yes	☐ no

15 Does the child retell short stories/fairy tales (using pictures)? ☐ yes ☐ no

Teil 3 - Communication

In the following you will read selected statements on children's communication behavior. In each case, please assess to what extent these behaviors apply to the child. Tick the correct statements for each answer. **Multiple ticking is possible** if the child shows several of the behaviors.

1. If you try to help the child with something they want to do on their own, e.g., getting dressed, how does the child react?

   ☐ The child cries and squirms.
   ☐ The child gets angry.
   ☐ The child pushes you away and tries to do it on her/his own.
   ☐ The child produces signs like "No!" or "Alone!".
   ☐ The child says something like: "Myself!", "Me!" or "Alone!".
   ☐ The child says something like, "I want to do this alone!".
   ☐ The child signs something like, "I want to dress myself!"
   ☐ The child uses a combination of signs and words to express herself/himself.

2. If you sit at the table and give the child something to eat that the child doesn't want, how does the child react?

☐ The child is crying.
☐ The child turns away or contorts the face.
☐ The child pushes the food away.
☐ The child shakes her/his head.
☐ The child produces the sign "No!".
☐ The child says: "No!".
☐ The child says something like: "Bah!", "Don't want to!" or "Don't like!".
☐ The child signs something like: "Disgusting!" or "Don't want to!"
☐ The child uses a combination of signs and words to make herself/himself understood.

3. If the child sees something that the child wants but can't get hold of, what does the child do?

☐ The child is crying.
☐ The child points to it.
☐ The child points to it, reaches for it and makes pleading noises.
☐ The child pulls you towards what it wants or pushes your hand in the right direction.
☐ The child looks or points at what it wants and produces a sign, e.g., the sign for "car" or "have".
☐ The child will show you a picture card with the corresponding icon, e.g., a car.
☐ The child calls you.
☐ The child looks or points to what it wants and says something like, "Car!" or "Have!".
☐ The child says something like: "I want the car.".
☐ The child signs something like, "I want the car."
☐ The child uses a combination of signs and words to make herself/himself understood.
☐ The child gets angry or distressed but does not ask for help.
☐ The child waits passively.

4. When the child is with other children, how does the child participate in play?

☐ The child plays alone.
☐ The child plays alongside the other children.
☐ The child watches the other children.
☐ The child takes adult guidance and involvement to participate.
☐ The child is talking to another child.
☐ The child signs with another child.
☐ The child uses a combination of signs and words to communicate.

5. If you ask the child to do something, e.g., "Please get your shoes!", what does the child do?

☐ The child shows no reaction.
☐ The child looks at you questioningly but doesn't seem to know what to do.
☐ The child does when it is part of a constant routine.
☐ The child will do what you ask if you show her/him the appropriate picture card.
☐ The child executes your request when you support your request with signs and pointing.
☐ The child carries out your request.

6. You and the children are walking along a road. Suddenly, the child sees something that interests her/him, such as an excavator. How does the child react?

☐ The child points to it.
☐ The child pulls or taps you and then points at it.
☐ The child points to it and says something like: "There!" or "Look!".
☐ The child points to it and produces a sign, e.g., the sign for “excavator”.
☐ The child says a word, e.g., "Excavator!".
☐ The child combines a word and a sign, e.g., the child says "There!" and produces the sign for " excavator".
☐ The child starts talking about it.
☐ The child starts signing about it.
☐ The child doesn't try to draw your attention to what is interesting.

Teil 4 – Speech intelligibility und speech comprehension

1. How intelligible is the child's speech to people who do not know them well?
   Give an approximate estimate in percentage, with 100% meaning "very well understood", 0% meaning "unintelligible" and e.g., 50% meaning "about half understandable". You can indicate any whole number from 1 to 100.

   ______%

2. How well does the child understand age-appropriate statements at a normal volume within a noise level that is normal in kindergarten groups?
   Give an approximate estimate in percentage, where 100% meanins "understands statements immediately", 0% means "does not understand statements" and e.g., 50% means "does not understand about half of the statements right away". Again, you may use all full numbers from 1 to 100 .

   _____%

Own observations/comments in connection with sign language in the kindergarten:

____

____

____

____

____

____

____

____

____

Thank you for your participation!

Supplemental Material B: Overview of demographic information for children in the inclusive groups from Schüler et al. (2021) and our co-enrollment group. The table provides information about gender, therapy, language, and SBE-3-KT score.

**Group**	**Gender**		**Therapy**		**Language**		**SBE-3-KT score**
	Girls	Boys	Yes	No	Mono-lingual	Bi-lingual	(Mean)
1 - Low implementation (n = 144)	63 (43.8%)	81 (56.2%)	27 (18.8%)	117 (81.2%)	79 (54.9%)	65 (45.1%)	141.7
2 - High implementation (n= 145)	70 (48.3%)	75 (51.7%)	31 (21.4%)	114 (78.6%)	85 (58.6%)	60 (41.4%)	136.1
3 - Co-enrollment (n = 11)	4 (36.3%)	7 (63.6%)	3 (27.3%)	8 (72.7%)	8 (72.7%)	3 (27.3%)	131.2

Supplemental Material C: The coding scheme used for video data analyses presented in this paper. This scheme was motivated by Preisler et al. (2002) and Schulz (2016). Please note that additional information in the videos were coded but not analyzed for the purpose of this paper and these categories are, therefore, not presented here.

	Class	Code	Explanations/Examples
1	**Child**	Code of the child in focus	
2	**Interaction partner**	Code of the child	Code of the direct interaction partner, whose interaction with the child in focus produced the interaction if this partner is a child.
		Code of the educator	Code of the direct interaction partner, whose interaction with the child in focus produced the interaction if the partner is an educator.
3	**Modality active**	Spoken language	Only spoken language is used, i.e., no signs used at all.
		Sign language	Only sign language is used, i.e., no spoken language used at all.
		Code-Blending	When spoken language and sign language are used at the same time (even if it occurs only once, i.e., a single sign accompanied by a spoken word in a longer stretch of signs or vice versa).
		Code-Switching	When switching from spoken language to sign language or vice versa.
		Nonverbal	If no lexicalized signs or words are used.
3a	**Additional modality active**	also Code-Blending	If spoken language and sign language are also used at the same time (even if it occurs only once).
		also Code-Switching	When switching also from spoken language to sign language or vice versa.
		No	If only one code applies in 3.

	Class	Code	Explanations/Examples
4	**Modality passive**	Spoken language	Only spoken language is used, i.e., no signs used at all.
		Sign language	Only sign language is used, i.e., no spoken language used at all.
		Code-Blending	When spoken language and sign language are used at the same time (even if it occurs only once, i.e., a single sign accompanied by a spoken word in a longer stretch of signs or vice versa).
		Code-Switching	When switching from spoken language to sign language or vice versa.
		Nonverbal	If no lexicalized signs or words are used.
4a	**Additional modality passive**	also Code-Blending	If spoken language and sign language are also used at the same time (even if it occurs only once).
		also Code-Switching	When switching also from spoken language to sign language or vice versa.
		No	If only one code applies in 4.
